# The Changing Landscape of Neuroscience Research, 2006–2015: A Bibliometric Study

**DOI:** 10.3389/fnins.2017.00120

**Published:** 2017-03-21

**Authors:** Andy Wai Kan Yeung, Tazuko K. Goto, W. Keung Leung

**Affiliations:** ^1^Oral and Maxillofacial Radiology, Applied Oral Sciences, Faculty of Dentistry, University of Hong KongHong Kong, Hong Kong; ^2^Department of Oral and Maxillofacial Radiology, Tokyo Dental CollegeTokyo, Japan; ^3^Periodontology, Faculty of Dentistry, University of Hong KongHong Kong, Hong Kong

**Keywords:** bibliometrics, cells, diagnostic imaging, functional neuroimaging, information science, literature-based discovery, neurosciences

## Abstract

**Background:** It is beneficial to evaluate changes in neuroscience research field regarding research directions and topics over a defined period. Such information enables stakeholders to quickly identify the most influential research and incorporate latest evidence into research-informed education. To our knowledge, no study reported changes in neuroscience literature over the last decade. Therefore, the current study determined research terms with highest citation scores, compared publication shares of research areas and contributing countries in this field from 2006 to 2015 and identified the most productive journals.

**Methods:** Data were extracted from Web of Science and Journal Citation Reports (JCR). Only articles and reviews published in journals classified under the JCR “Neurosciences” category over the period of interest were included. Title and abstract fields of each included publication were extracted and analyzed via VOSviewer to identify recurring terms with high relative citation scores. Two term maps were produced for publications over the study period to illustrate the extent of co-occurrence, and the impact of terms was evaluated based on their relative citation scores. To further describe the recent research priority or “hot spots,” 10 terms with the highest relative citation scores were identified annually. In addition, by applying Bradford's law, we identified 10 journals being the most productive journals per annum over the survey period and evaluated their bilbiometric performances.

**Results:** From 2006 to 2015, there were 47 terms involved in the annual lists of top 10 terms with highest relative citation scores. The most frequently recurring terms were autism (8), meta-analysis (7), functional connectivity (6), default mode network (4) and neuroimaging (4). Neuroscience research related to psychology and behavioral sciences showed an increase in publication share over the survey period, and China has become one of the major contributors to neuroscience research. Ten journals were frequently identified (≥8 years) as core journals within the survey period.

**Discussion:** The landscape of neuroscience research has changed recently, and this paper provides contemporary overview for researchers and health care workers interested in this field's research and developments. Brain imaging and brain connectivity terms had high relative citation scores.

## Introduction

Neuroscience is an exciting research field, and many recent discoveries have informed paradigm-shifts and innovations. For instance, the 2014 Nobel Prize in Physiology or Medicine was awarded to Professors John O'Keefe, May-Britt Moser and Edvard Moser, neuroscientists who discovered place cells and grid cells that form a built-in, global positioning system inside the brain (O'keefe and Burgess, 2005; Moser et al., 2008). Indeed, neuroscience research has received substantial support across the globe. In the United States, the Human Connectome Project was launched in 2009 (Battery, 2010), followed by the Brain Research through Advancing Innovative Neurotechnologies (BRAIN) Initiative in 2013 (Insel et al., 2013). Concurrently, the Human Brain Project was initiated in Europe (Markram, 2012). In Japan, the Brain Mapping by Integrated Neurotechnologies for Disease Studies (Brain/MINDS) program started in 2014, and the Brain Science and Brain-Like Intelligence Technology project will soon be officially launched by China, though significant preliminary research has already been done (Grillner et al., 2016). With these abundant investments in neuroscience in recent years, more explorative, fundamental or basic research could be conducted, which eventually led to today's paradigm shifts—or paradigm shifts yet to come. For instance, the emergence of brain connectivity studies (Friston, 2011) in recent years may enable us to compare and contrast the normal and diseased brains, and thus identify abnormal brain connectivity patterns as potential biomarkers for neurologic diseases. These advances in the neuroscience field not only drive significant progress in brain biology but also confer practical implications to medicine. We should evaluate and track the changes regarding the landscape of neuroscience research, to indicate evolution regarding research directions, health care priorities and prime translational research topics over a defined period. Such information enables stakeholders to clearly and quickly identify the most influential research works and incorporate the latest evidence into research-informed education.

Bibliometric studies could provide relevant evaluations and assessments of the social and scientific relevance of a specific discipline or research field of interest (López-Muñoz et al., 2006, 2014). There have been numerous bibliometric studies on the performance of neuroscience research outputs in specific countries, including China (Xu et al., 2003), India (Shahabuddin, 2013) and Sweden (Glänzel et al., 2003). In addition, similar studies have been conducted in relevant research fields such as psychiatry and psychopharmacology (López-Muñoz et al., 2006, 2014). However, to our knowledge, no published study has reported overall changes in the landscape of neuroscience literature across the globe over the last decade.

Therefore, the aims of this study was to identify the most productive core journals in the field from 2006 to 2015, determine the research terms with the highest citation impact, and compare the publication shares of research areas and contributing countries across these 10 years.

## Materials and methods

### Date source

Data analyzed in this study were extracted from Web of Science (WoS) and Journal Citation Reports (JCR). Both required a subscription and were hosted by Thomson Reuters.

### Data extraction

Publications indexed in WoS were included if they were: (1) articles or reviews; (2) published in journals under the JCR “Neurosciences” category; and (3) published from 2006 to 2015. Full records and cited references of the included publications were manually downloaded. The analyses at the literature level (term maps, annual high-impact terms and changes in research areas and contributing countries) were based on all these records; and for each publication, citations were counted until the end of November, 2016.

### Term maps

For each year, the title and abstract fields of each included publication were extracted and analyzed via VOSviewer (van Eck and Waltman, 2009). Two term maps, one for 2006 and one for 2015, were produced to illustrate a network of recurring keywords, showing their co-occurrence and relative citation impacts. The details of the established methods and algorithms have been described in previous publications (van Eck et al., 2010; Waltman et al., 2010). In brief, for each term map, only terms that occurred at least 100 times under binary counting were considered. Note that binary counting only considers the number of publications in which a term was present; it disregards the number of occurrences of a term within a single publication. General noun phrases were removed using an algorithm (van Eck and Waltman, 2011). Of the remaining terms, 500 with the highest relevance score calculated via VOSviewer were used to form a term map to allow for network visualization. The algorithm was designed to ensure that terms that co-occurred more frequently were positioned closer to each other. Terms that occurred more frequently had larger bubbles. Before the final map was created, we visually inspected the map, removed irrelevant terms and combined the abbreviated forms of the noun phrases (Heersmink et al., 2011). Depending on the citation counts of the respective publications, each term received a relative citation score (beginning at zero) that is represented by color. Blue (0) indicates below average, green (1) indicates average and red (≥2) indicates above average. The coloring scheme was based on previous publications (van Eck et al., 2013; Waltman et al., 2014).

### Annual high-impact terms

For each year, we examined terms that occurred at least 100 times. We then used VOSviewer to calculate a relative citation score for each term based on citation counts. Normalized citation score based on average citation counts is one of the key concepts in bibliometrics (Waltman, 2016). The calculation was performed as follows. For each year, each publication was given a normalized citation score by dividing its number of citations (received in that year) by the average number of citations of all publications (received in that year) of the neuroscience field. A score of 1 implies the citation count of that particular publication equals the average of all publications appearing in neuroscience field in that year. Subsequently, each term received its own relative citation score by averaging the normalized citation scores of all publications in which the term occurred in the title or abstract. Previous studies have documented the details of this automated procedure to identify terms with higher-than-average citation scores (van Eck et al., 2013; Waltman et al., 2014). We identified 10 relevant terms with the highest relative citation score (highest impact) for each year in the survey period. Their relative citation scores were tracked annually.

### Growth of publication

Price's Law, one of the most widely utilized indicators for analyzing the growth of publication within a specific research field, which was regularly applied to evaluate whether publication in a research field has experienced an exponential growth over a pre-defined survey period (López-Muñoz et al., 2006, 2014), was utilized to assess the annual change in publication count in neuroscience journals in the current study. After plotting the annual publication count (y) against year (x), we applied the best-fitting linear and exponential trend lines to the plot and recorded the mathematical equations forming the trend lines. If the data fitted better to the exponential line than the linear line, results will be considered as fulfilling the Price's Law.

### Changes in research areas and contributing countries

We identified the research areas and contributing countries of the publications in 2006 and 2015, the first and last year of the study period, respectively. These data were obtained by analyzing the search results in WoS. In WoS, publications (according to the journals where they were published) were classified into over 250 research areas in which each area contains papers similar in scope and citation characteristics^1^. To illustrate the relationship with social-health parameters, the relationship between the publication shares of the most productive countries and their respective total per capita expenditure on health^2^ were evaluated, as described in previous bibliometric studies (López-Muñoz et al., 2006, 2008a, 2014).

### Core journals under Bradford's law and their performance

For each year, Bradford's law of scattering was applied to identify the core journals in the neuroscience category. The core journals identified for each year are those that contributed one-third of total publications for that year (Vickery, 1948). To evaluate the bibliometric performance of the top 10 core journals (the 10 journals with highest frequency of occurrence in the annual core journal lists over the survey period), time trends of their Impact Factor (IF), Immediacy Index (II), and Eigenfactor Score (ES) were evaluated. IF indicates the yearly average citation count of its recent publications, II refers to the average citation count of current-year publications and ES, though similar to IF, weights citations according to the ranking of the citing journals. The time trends of IF, II, and ES were evaluated by separate linear regressions, with year as the independent variable (López-Abente and Muñoz-Tinoco, 2005; Jayaratne and Zwahlen, 2015). The slope, β, indicated the average magnitude of change over time, and *R*^2^ indicated the fitness of the regression model. Statistical analyses were performed with SPSS 23.0 (IBM, New York, USA). Results were significant if *P* < 0.05.

## Results

From 2006 to 2015, a total of 340,210 publications matched with the selection criteria of this study (all articles and reviews in JCR “Neurosciences” category), of which 305,175 were articles and 35,035 were reviews. The marked annual increase in the number of neuroscience publications from 2006 to 2015 followed a linear trend more closely than an exponential curve (Supplementary Figure 1). The linear fitting of the data obtained a coefficient of determination of *r*^2^ = 0.9959, whereas the exponential fitting only obtained a coefficient of determination of *r*^2^ = 0.9914.

### Term maps

**Figure 1:**
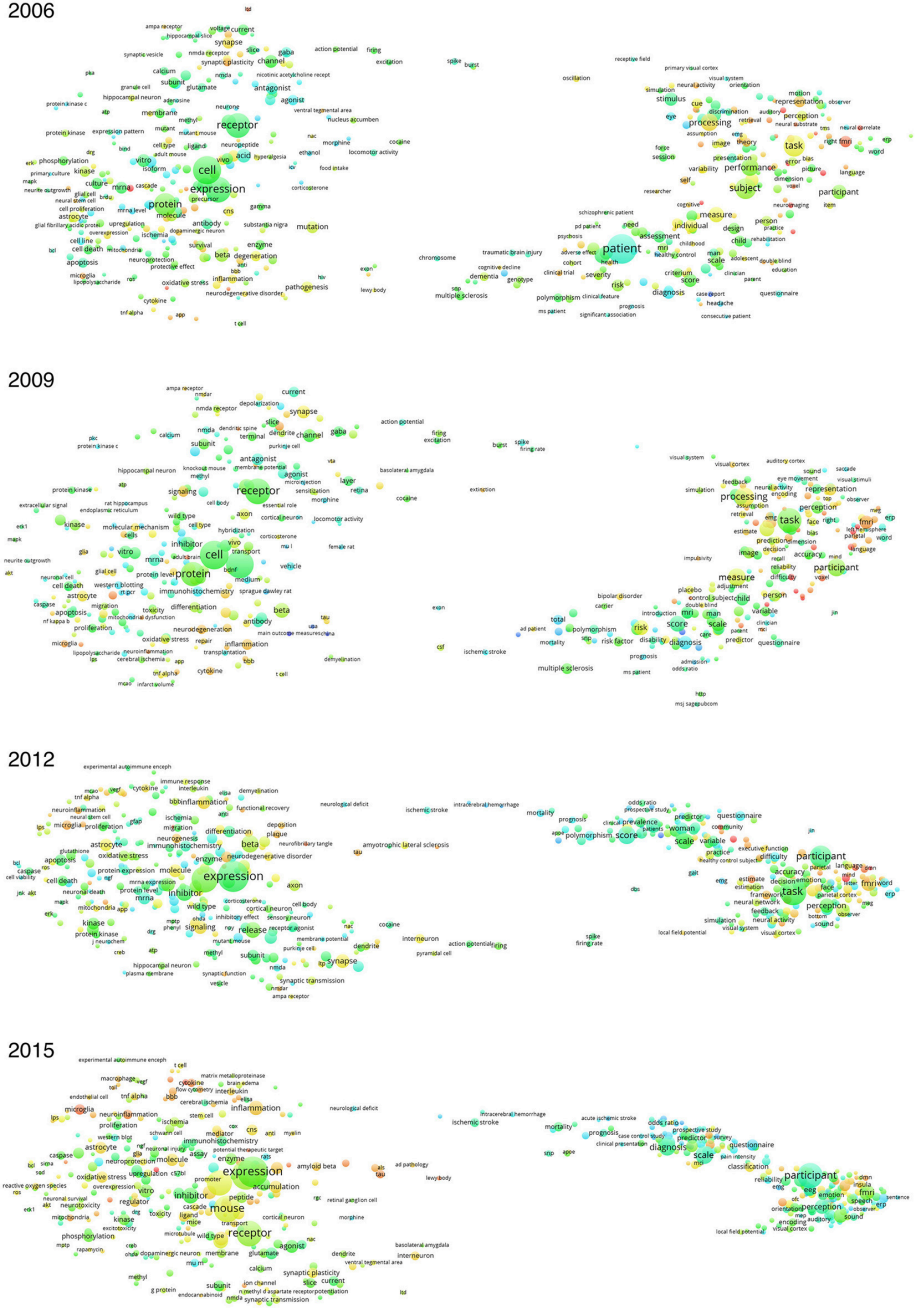
**Term maps for neuroscience literature**. In general, terms on the left are more related to cellular, molecular or genetic neuroscience, whereas terms on the right are more related to brain imaging. Bubble size is related to the occurrence of terms in the titles and abstracts in the included publications. Color is related to relative citation impact attributable to the terms, with blue indicating below average, green indicating average and red indicating above average. Moving from 2006 to 2015, it could be observed that the terms on the left were gaining relative citation impact compared to the terms on the right. Certain terms remained having relatively high citation scores, such as inflammation, microglia, mitochondria and tau (colored red and orange). Some remained having relatively low citation scores, such as ischemic stroke, intracerebral hemorrhage and mortality.

The term maps for 2006 and 2015 are shown in Figure 1. For both maps, terms on the left are more related to cellular, molecular or genetic neuroscience research using animal models, such as cell, protein, receptor, expression and mice. Terms on the right are more related to brain imaging involving humans, such as functional magnetic resonance imaging (fMRI), perception, task, performance and patient. For 2006, the red and orange bubbles are more concentrated on the right side of the map, e.g., fMRI and neuroimaging. For 2015, the red and orange bubbles are more concentrated on the left side of the map, e.g., microglia and neuroinflammation. Across the survey period, some of the basic terms remained red and orange, such as inflammation, microglia, mitochondria and tau. Clinical terms remained blue and green, such as mortality, multiple sclerosis, intracerebral hemorrhage and ischemic stroke.

### Annual high-impact terms

Ten relevant terms with highest impact were identified for each year from 2006 to 2015; they are listed in Table 1. There were 47 terms over the survey period (Table 1). The most frequently recurring term was autism (top 10 in 8 years out of 10). Next were meta-analysis (7 years out of 10), functional connectivity (6 years out of 10), default mode network (4 years out of 10) and neuroimaging (4 years out of 10). Over the last 3 years (2013–2015), three terms appeared on the top 10 list: melatonin, microglia and neurofibrillary tangle.

**Table 1:** **Relative citation scores of recurring top 10 high impact terms (bold) from 2006 to 2015**.

**Term**	**2006**	**2007**	**2008**	**2009**	**2010**	**2011**	**2012**	**2013**	**2014**	**2015**
Autism	**2.05**	**2.17**	**2.68**	**1.99**	**1.99**	**2.42**	**2.11**	1.60	**1.93**	1.59
Cognitive control	NA	NA	NA	NA	**2.22**	**2.37**	**1.83**	1.66	1.47	1.23
Decision making	NA	**2.40**	1.72	**1.88**	**1.94**	1.66	1.35	1.23	1.15	1.20
Default mode network	NA	NA	NA	NA	NA	**2.52**	**2.28**	**1.82**	**1.80**	1.62
Diffusion tensor imaging	NA	**2.35**	**2.36**	**2.44**	1.77	1.76	1.56	1.28	1.34	1.14
Functional connectivity	NA	NA	**3.05**	**2.56**	**2.79**	**2.37**	**2.15**	**1.86**	1.74	1.47
Insula	NA	**2.12**	NA	**2.01**	**2.21**	1.72	1.75	1.62	1.38	1.32
Melatonin	0.76	0.90	0.94	1.07	1.19	1.39	1.35	**1.85**	**1.87**	**2.17**
Meta-analysis	NA	**1.98**	**3.30**	**3.10**	**2.61**	**2.34**	**2.26**	**1.86**	1.72	1.53
Microglia	1.58	1.88	1.56	1.57	1.58	1.39	1.54	**1.87**	**1.88**	**1.77**
MicroRNA	NA	NA	NA	NA	NA	NA	**1.81**	**1.91**	**1.82**	1.55
Neurofibrillary tangle	1.16	NA	NA	NA	1.48	1.50	1.77	**1.86**	**1.86**	**1.74**
Neuroimaging	**1.97**	1.51	1.66	**1.96**	1.71	**1.95**	**1.83**	1.43	1.74	1.54
Orbitofrontal cortex	NA	**2.05**	**2.02**	1.72	**2.12**	1.70	1.60	NA	1.39	1.34
Systematic review	NA	NA	NA	NA	**1.94**	**1.92**	**1.90**	1.52	1.60	1.55

*NA, not available. Only terms that occupied a place in the annual top 10 list thrice or more over 2006–2015 were listed (n = 15). Other terms (n = 32) that were in the annual top 10 list ≤ 2 times over 2006–2015 included alpha synuclein, Alzheimer's disease, anterior cingulate cortex, astrocyte, autophagy, blood-oxygen-level dependent signal, brain function, brain network, cognition, drug addiction, emotion, executive function, fractional anisotropy, immune system, ketamine, Lewy body, major depressive disorder, medial prefrontal cortex, mild cognitive impairment, mind, mitochondrial dysfunction, morphometry, neurogenesis, obesity, oxytocin, posterior cingulate cortex, potential therapeutic target, rapamycin, social interaction, transcranial direct current stimulation, ventral striatum and voxel*.

### Research areas with largest publication shares in 2006 and 2015

In 2006, the most productive research area was psychiatry, accounting for nearly one-tenth of relevant publications (2,762/29,379, Table 2). In 2015, its share reduced to 7.35% (2,852/38,792) apparently as a result of 32.0% increase in total publication. It was replaced by psychology (3,076/38,792) as the most productive research area. By 2015, ophthalmology and zoology no longer occupied a place on the list, and they were replaced by immunology and “geriatrics, gerontology.” In fact, the publication share of “geriatrics, gerontology” in 2015 (676/38,792) was 1.74%, two times of that in 2006 (205/29,379).

**Table 2:** **Top 10 research areas with largest publication shares in 2006 and 2015**.

**Research area**	**2006 rank**	**% of share**	**2015 rank**	**% of share**	**Differences in % share**
Psychiatry	1	9.40	3	7.35	−2.05
Physiology	2	6.69	5	4.89	−1.80
Behavioral sciences	3	6.43	2	7.77	+1.34
Pharmacology, pharmacy	4	6.39	4	7.20	+0.81
Biochemistry, molecular biology	5	6.18	6	4.48	−1.70
Psychology	6	5.72	1	7.93	+2.21
Endocrinology, metabolism	7	3.10	7	3.24	+0.14
Radiology, nuclear medicine, medical imaging	8	3.03	8	3.06	+0.03
Ophthalmology	9	1.82	26	0.71	−1.11
Zoology	10	1.69	27	0.71	−0.98
Immunology	12	1.28	9	1.92	+0.64
Geriatrics, gerontology	20	0.70	10	1.74	+1.04

*Research areas were defined by Web of Science hosted by Thomson Reuters*.

### Most productive countries

The United States remained the leading country in neuroscience research output over the 10-year survey period. Despite a reduction in its publication share, the United States still accounted for 37% (14,279/38,792) of total publications and was the largest contributor in 2015. European countries, such as Germany and the United Kingdom, were also major contributors to the field. One country with a substantial increase in publication share was China: Between 2006 and 2015, its publication share in this field tripled (2006: 870/29,379 = 2.96%, vs. 2015: 4,358/38,792 = 11.23%, Table 3). With regard to social-health data, it seemed that the publication shares of the most productive countries were directly proportional to their respective total per capita expenditure on health (Supplementary Figure 2). However, if data from China and United States were excluded, the relationship became much less apparent.

**Table 3:** **Top 10 most productive neuroscience research countries in 2006 and 2015**.

**2015**	**2006 rank**	**% of share**	**2015 rank**	**% of share**	**Differences in % share**
USA	1	41.05	1	36.81	−4.24
Germany	2	9.93	3	10.20	+0.27
UK	3	8.63	4	8.75	+0.12
Japan	4	8.36	7	5.69	−2.67
Canada	5	6.47	5	6.88	+0.41
Italy	6	5.91	6	6.49	+0.58
France	7	5.48	8	4.99	−0.49
Netherlands	8	3.42	10	4.20	+0.78
Australia	9	3.39	9	4.54	+1.15
Spain	10	3.23	11	3.62	+0.39
China	11	2.96	2	11.23	+8.27

*Each country had one count if it appears in the author affiliations field of a publication, as counted by Web of Science hosted by Thomson Reuters*.

### Identification of core journals under Bradford's law

The number of neuroscience journals increased from 200 in 2006 to 256 in 2015. In parallel, the number of core journals doubled from 11 (11/200 = 5.5%) in 2006 to 22 (22/256 = 8.6%) in 2015 (Table 4). Six journals were consistently included as core journals throughout 2006–2015, namely *Brain Research, Journal of Neurophysiology, Journal of Neuroscience, NeuroImage, Neuroscience*, and *Neuroscience Letters*. Together with four other journals, namely *Behavioural Brain Research* (9 years), *Experimental Brain Research* (9 years), *European Journal of Neuroscience* (8 years), and *Journal of the Neurological Sciences* (8 years), they formed the top ten core neuroscience journals. The complete list of 33 core neuroscience journals is presented in Supplementary Table 1.

**Table 4:** **Division of neuroscience journals and publications from 2006 to 2015 according to Bradford's law**.

	**Journal number (publication count)**			
**Year**	**Zone 1: core**	**Zone 2**	**Zone 3**	**Total**
2006	11 (8,901)	38 (8,989)	151 (9,267)	200 (27,157)
2007	13 (9,472)	40 (9,412)	158 (9,550)	211 (28,434)
2008	15 (9,839)	43 (9,918)	163 (9,838)	221 (29,595)
2009	16 (10,283)	47 (10,252)	168 (10,016)	231 (30,551)
2010	16 (10,614)	49 (10,770)	174 (10,804)	239 (32,188)
2011	18 (11,131)	51 (11,151)	175 (11,029)	244 (33,311)
2012	19 (11,466)	53 (11,449)	180 (11,517)	252 (34,432)
2013	21 (11,818)	54 (11,912)	177 (11,789)	252 (35,519)
2014	21 (12,223)	54 (12,273)	177 (12,367)	252 (36,863)
2015	22 (12,388)	53 (12,392)	181 (12,539)	256 (37,319)

*According to Bradford's law, journals were divided into three zones, and each zone should contain one-third of total publication count. Zone 1 journals were identified as core journals. Publication count here refers to the total count of articles and reviews*.

### Bibliometric performance of core journals

**Figure 2:**
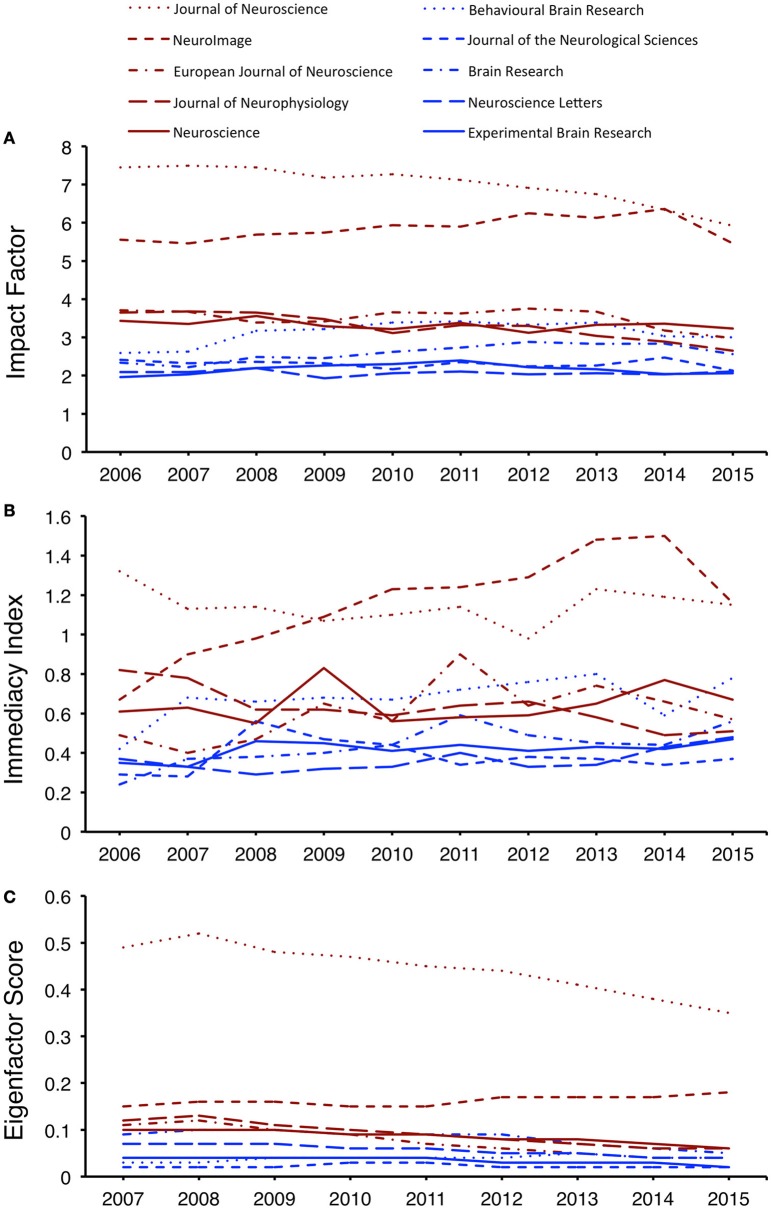
**Bibliometric performances of the key core journals. (A)** Time trends of Impact Factor. **(B)** Time trends of Immediacy Index. **(C)** Time trends of Eigenfactor Score.

In general, the core journals maintained their performance levels over the survey period. Most (7/10) core journals did not exhibit a significant change in IF from 2006 to 2015 (Figure 2A). One journal, *Brain Research*, had a significantly improved IF (β = 0.06, *P* < 0.01), whereas two journals experienced a significant decrease in IF, namely *Journal of Neurophysiology* (β = −0.11, *P* < 0.001) and *Journal of Neuroscience* (β = −0.16, *P* < 0.001).

Six of the core journals did not have a significant change in II over the survey period (Figure 2B). Three journals had significantly improved II, namely *Brain Research* (β = 0.03, *P* = 0.01), *NeuroImage* (β = 0.07, *P* < 0.01) and *Neuroscience Letters* (β = 0.01, *P* = 0.05). One journal, *Journal of Neurophysiology*, had a decreased II (β = −0.03, *P* < 0.01).

In terms of ES, most (7/10) core journals were subject to a decrease, and only two an increase (Figure 2C). However, these changes were small (β = −0.02–0.00).

## Discussion

Using data from WoS and JCR, the results of the current study revealed transformations in the field of neuroscience research from 2006 to 2015 in terms of leading or core journals, distribution of publications, research context and contributions by country. No attempt was made to retrospectively analyze data earlier than 2006, because this study's focus only concerns recent bibliometric growth rather than the complete history of this research field. The annual growth of neuroscience publications over the survey period indicated non-fulfillment of Price's Law, i.e., no exponential growth was observed. It experienced a linear growth instead. This linear increment was consistent with publications in adjacent research fields such as psychiatry (López-Muñoz et al., 2008b) and psychopharmacology (López-Muñoz et al., 2013). On the other hand, there was an exponential increase in publications in melatonin (López-Muñoz et al., 2016), bipolar disorder (López-Muñoz et al., 2006) and attention-deficit hyperactivity disorder (López-Muñoz et al., 2008a). Since the current study aimed at providing an overview of the neuroscience literature, we did not apply Lotka's Law to evaluate the productivity of individual researchers.

### Shifting of citation focus as seen from term maps

The term maps from Figure 1 illustrated the shift of citation focus from general brain imaging terms to cellular, molecular and genetic terms. In recent years, molecular imaging of the brain is gaining attention, such as that related to neuroinflammation of microglia and macrophages (Venneti et al., 2013) and neurodegeneration related to tau protein and Lewy body (Wakabayashi et al., 2013). These research topics investigated in experimental animal models probably led to the increased relative citation impacts of these terms. Certain clinical topics remained having low citation impacts, such as multiple sclerosis, intracerebral hemorrhage and ischemic stroke.

### Brain imaging terms as recurring high-impact terms

Results in Table 1 demonstrated that brain imaging terms occupied three of the top five recurring high-impact terms, namely default mode network, functional connectivity and neuroimaging. The rise in relative citation impact of brain connectivity terms was consistent with the launches of several research funds, including the aforementioned BRAIN Initiative, Human Brain Project and Human Connectome Project. There were two other terms related to brain connectivity among the top 10 highest impact terms (diffusion tensor imaging and fractional anisotropy). One popular research topic was to investigate the co-activation (also known as functional connectivity) of different brain regions (Yeung et al., 2017b). One such network of co-activation during a resting state is called the default mode network, which is believed to be robust across populations, and, as a result, it has gained attention in recent years (Raichle and Snyder, 2007). Another area that merits mention is the advancement in the use of statistics, such as the implementation of dynamic causal modeling to neuroimaging data in 2003 (Friston et al., 2003), which have enabled an inference of effective connectivity. This application illustrates the directionality of communications between brain regions during particular experimental tasks (Nakamura et al., 2013; Yeung et al., 2016). Though the terms related to effective connectivity were not in the annual top 10 lists of high-impact terms during the survey period, they may well be a potential hot spot in the field in the future (Friston, 2011).

### The high impact of terms related to Alzheimer's disease over the last 3 years

As reported, three terms always appeared on the top 10 list of high-impact terms over the last 3 years, namely melatonin, microglia and neurofibrillary tangle. Collectively, these three terms are related to Alzheimer's disease. Evidence has suggested that Alzheimer's disease, with intracellular neurofibrillary tangle a widely recognized biomarker, is linked to neuroinflammatory changes caused by overactivation of microglia that can be alleviated by administration of melatonin (Smith et al., 2012; Lin et al., 2013). This effect of melatonin may be related to regulation of autophagy (Choi et al., 2013). Moreover, we identified *Journal of Alzheimer's Disease* as one of the core journals (2010–2015) in the current study. Together, these findings suggest that topics relevant to this disease have recently become high impact.

### Attention to autism

Autism appeared in the annual list of top 10 high-impact terms eight times in the 10-year survey period. On average, its relative citation score was 2.05 (citation count 2.05 times that of an average term), outperforming the relative citation score of other neurological disorders such as major depressive disorder (1.68) and mild cognitive impairment (1.33).

### Research directions, health care priorities and prime translational research topics

Results of the current bibliometric study may help identify three important areas of neuroscience research: research directions, health care priorities and prime translational research topics. First, with regard to research directions, it could be observed that brain imaging was one of the highest impact over the last decade. Looking at health care priorities, as it was demonstrated that meta-analysis was a high-impact term in neuroscience field, researchers may consider conducting more meta-analyses to answer priority questions for better evidence-based practices in health care industry or health care services. As for prime translational research topics, brain connectivity had high impact; thus, its aberrations among patients with various neurodegenerative or neurological disorders can now be further investigated.

### Increased publication share of geriatrics and gerontology

The publication share of geriatrics and gerontology doubled from 2006 to 2015. This was reasonably expected due to the aging population of the developed countries. As the elderly people are more prone to neurodegenerative and neuroinflammatory diseases such as Alzheimer's disease (Clegg et al., 2013), the citation impact of terms relevant to neurodegeneration and neuroinflammation also increased over the study period.

### The United States and European countries as major contributors

Despite changes in research hot spots, the most productive or research-leading countries did not change significantly between 2006 and 2015. Developed countries, mainly ones in the Western world, were still major contributors to neuroscience research, i.e., Australia, Canada, France, Germany, Japan, Netherlands, Italy, the United Kingdom and the United States. Incidentally, these countries were also previously identified as major contributors to various topics within or relevant to neuroscience, such as neuroimaging (Kim et al., 2016; Yeung et al., 2017a) and Parkinson's disease (Li et al., 2008), which means these countries may have relatively more established research backgrounds and research funding. However, with more economic development and financial input into academic research, China has emerged as one of the important contributors in neuroscience research field in recent years. The correlation of publication share in neuroscience with the per capita health expenditure of the most productive countries might be consistent with the notion that the higher the spending on health, the more the research publication (López-Muñoz et al., 2006, 2014). However, it should be interpreted with caution because data from China and the United States were relatively influential as potential outliers.

### Growth in publication and stability of key core journals identified by Bradford's law

The annual publication (articles and reviews) count of neuroscience research increased every year, as did the number of neuroscience journals. By applying Bradford's law of scattering, we revealed that the percentage of journals classified as core journals likewise increased over the study years. These changes indicated that this field has been growing and becoming more dispersed. Besides, the bibliometric performances of the key core journals were mostly stable over the study period.

### Implications and future perspectives

The shift of citation focus as seen from term maps and Table 1 indicated the importance of co-existing animal models and *in vitro* studies together with human neuroimaging studies. While neuroimaging studies may continue to unveil the brain function or neural mechanism of relevant physiological processes, animal models and *in vitro* studies may also be required to explore and explain the details down to genetic, cellular or molecular levels.

Moreover, with the increasing publication count and wider distribution of publications among neuroscience journals, future publications should devise keywords, titles and abstracts more thoughtfully. Search strategy of fellow researchers should be taken into careful consideration so that the relevant works can be identified/retrieved from search engines or databases.

By considering the specific hot topics having high relative citation scores altogether, future work may focus on utilizing brain imaging and connectivity analyses to identify biomarkers that may signify neurodegenerative or neuroinflammatory tendency among the aging population.

### Study limitations

This study has certain limitations. First, this study is retrospective, and, though it tracked past or recent trends, rapidly developing interests in a short (recent) period may not be accurately reflected. Second, the work examined in this study consists exclusively of articles and reviews published from 2006 to 2015 in JCR “Neurosciences” journals as indexed by WoS. It may not be sufficient to represent all neuroscience literature, such as other document formats published in journals or publications not included in this study. As neuroscience interacts with many other academic fields, some arguably relevant studies may have been published in journals not classified as neuroscience journals. However, it would be difficult to define search terms that effectively cover all papers touching on neuroscience across all scientific literature while simultaneously excluding irrelevant ones. Therefore, we focused on JCR “Neurosciences” journals. Third, the analyses in this study were based on data recorded in the WoS database. Although Google Scholar and other databases may offer broader coverage, much of this “extra” coverage may be attributed to journals with potentially limited audience. Given that we aimed to determine research changes in the core of the neuroscience community, we followed previous neuroscience bibliometric publications by only accessing WoS (Glänzel et al., 2003; Li et al., 2008; Bishop, 2010). Therefore, the results from the current study should be interpreted with considerations that journals of other sections of JCR (pharmacology, diagnostic imaging, biochemistry, biology, etc.) were not included. Future studies may expand to them and consider the access to other databases such as PubMed/Medline, Scopus or Embase when deemed appropriate. Besides VOSviewer, future studies may consider using other bibliometric tools to further dissect the evidence, such as using Pajek to analyze the citation and co-authorship networks of selected data subsets (Batagelj and Mrvar, 1998), and using Publish or Perish^3^ to evaluate the distribution of citations received by selected authors in terms of h-index or g-index.

## Conclusions

To conclude, our findings revealed changes in the landscape of neuroscience research over the study period and provided a contemporary overview of neuroscience research for researchers and health care workers interested in this field. Brain imaging and brain connectivity have been shown to be hot topics, and Alzheimer's disease and associated topics have recently gained traction.

## Author contributions

AY conceived the work, acquired and analyzed data and drafted the work. TG and WK facilitated the acquisition of data and critically revised the work. All authors have approved the final content of the manuscript.

## Funding

This work was substantially supported by a grant from the Research Grants Council of the Hong Kong Special Administrative Region, China (HKU 766212M).

### Conflict of interest statement

The authors declare that the research was conducted in the absence of any commercial or financial relationships that could be construed as a potential conflict of interest.

## Footnotes

^1^http://ipscience-help.thomsonreuters.com/inCites2Live/filterValuesGroup/researchAreaSchema.html

^2^Data available from http://www.who.int/countries/en/

^3^Harzing, A. W. (2007) *Publish or Perish*. Available online at: http://www.harzing.com/pop.htm

## Supplementary material

Supplementary Figure 1 **Annual growth of neuroscience publication from 2006 to 2015**. After plotting the annual publication count (y) against year (x), we applied the best-fitting linear (solid) and exponential (dash) trend lines to the plot. Results indicated the growth followed a linear trend (y = 1168.8x–2 × 10^6^, *r*^2^ = 0.9959) more closely than an exponential trend (y = 7 × 10^−28^e^0.0363*x*^, *r*^2^ = 0.9914).

Supplementary Figure 2 **The relationship between the neuroscience publication share and the total per capita expenditure on health (USD$) among the most productive countries as surveyed in 2006 and 2015**. The economic data was obtained from World Health Organization (http://www.who.int/county/en/). It seemed that the publication shares of the most productive countries were directly proportional to their respective total per capita expenditure on health (2006: *r*^2^ = 0.560, *p* = 0.005; 2015: *r*^2^ = 0.352, *p* = 0.054). However, if data from China and United States were excluded, the relationship became much less apparent (2006: *r*^2^ = 0.008, *p* = 0.817; 2015: *r*^2^ = 0.010, *p* = 0.800). AUS, Australia; CAN, Canada; CHI, China; FRA, France; GER, Germany; ITA, Italy; JAP, Japan; NET, Netherlands; SPA, Spain; UK, United Kingdom; USA, United States.

Supplementary Table 1 **List of core neuroscience journals from 2006 to 2015**. A black dot denoted that journal belonged to the core journals (assigned to Zone 1 according to Bradford's law) in that year. NA, non-applicable because that journal did not exist in that year. Core journals are the most productive journals; together they contributed to one-third of total count of articles and reviews in the census year (Table 4).
