# Supplementary figures and images for: The Changing Landscape of Neuroscience Research, 2006–2015: A Bibliometric Study
Source: Front Neurosci. 2017 Mar 21;11:120. doi: 10.3389/fnins.2017.00120 (PMC5360093; doi:10.3389/fnins.2017.00120)

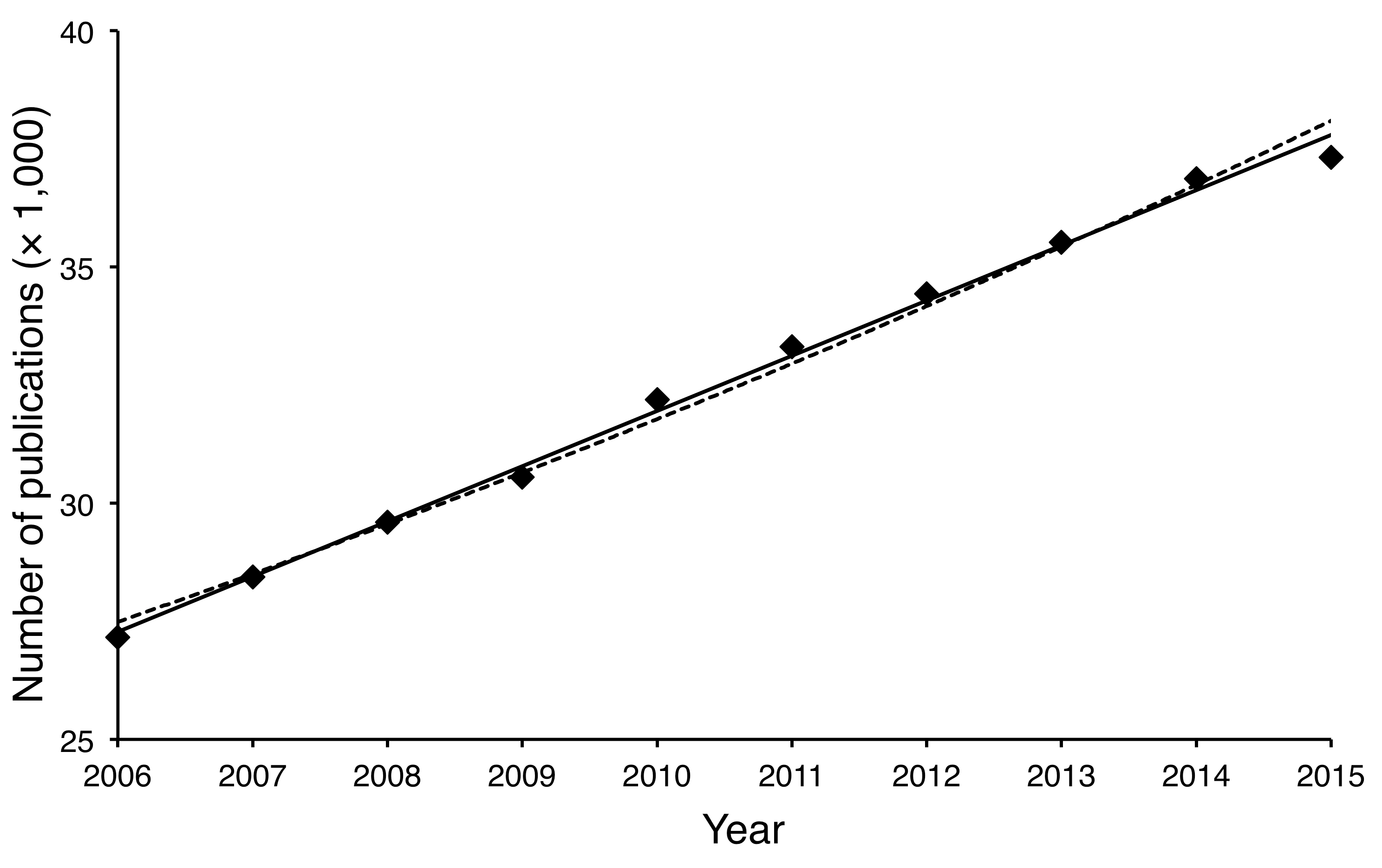

Supplement: Supplementary Figure 1 — Annual growth of neuroscience publication from 2006 to 2015. After plotting the annual publication count (y) against year (x), we applied the best-fitting linear (solid) and exponential (dash) trend lines to the plot. Results indicated the growth followed a linear trend (y = 1168.8x–2 × 10^6^, r2 = 0.9959) more closely than an exponential trend (y = 7 × 10^−28^e0.0363x, r2 = 0.9914). [file Image1.TIFF]

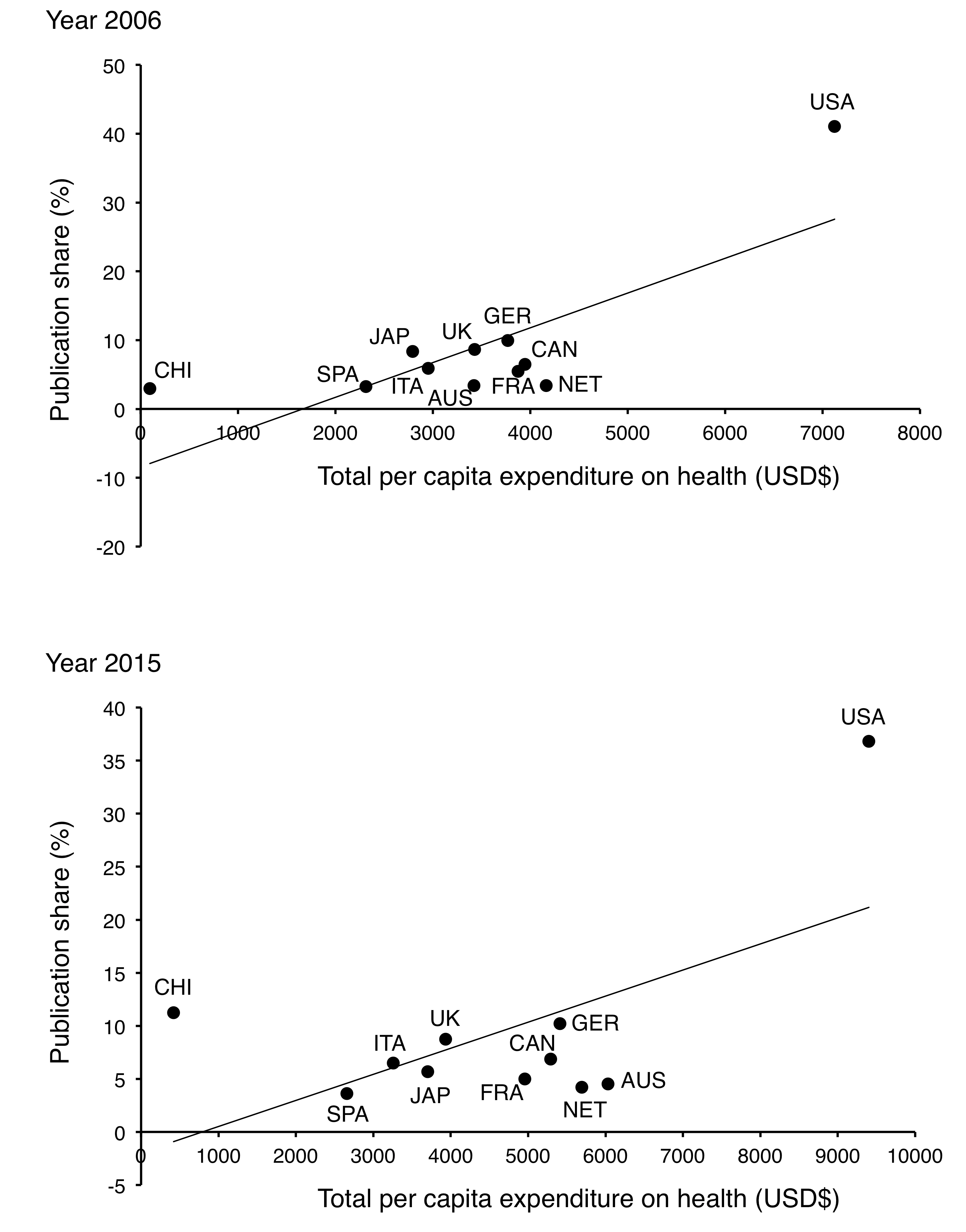

Supplement: Supplementary Figure 2 — The relationship between the neuroscience publication share and the total per capita expenditure on health (USD$) among the most productive countries as surveyed in 2006 and 2015. The economic data was obtained from World Health Organization (http://www.who.int/county/en/). It seemed that the publication shares of the most productive countries were directly proportional to their respective total per capita expenditure on health (2006: r2 = 0.560, p = 0.005; 2015: r2 = 0.352, p = 0.054). However, if data from China and United States were excluded, the relationship became much less apparent (2006: r2 = 0.008, p = 0.817; 2015: r2 = 0.010, p = 0.800). AUS, Australia; CAN, Canada; CHI, China; FRA, France; GER, Germany; ITA, Italy; JAP, Japan; NET, Netherlands; SPA, Spain; UK, United Kingdom; USA, United States. [file Image2.TIFF]
