# Supplementary material for: The Changing Landscape of Neuroscience Research, 2006–2015: A Bibliometric Study
Source: Front Neurosci. 2017 Mar 21;11:120. doi: 10.3389/fnins.2017.00120 (PMC5360093; doi:10.3389/fnins.2017.00120)
Supplement: Supplementary Table 1 — List of core neuroscience journals from 2006 to 2015. A black dot denoted that journal belonged to the core journals (assigned to Zone 1 according to Bradford's law) in that year. NA, non-applicable because that journal did not exist in that year. Core journals are the most productive journals; together they contributed to one-third of total count of articles and reviews in the census year (Table 4). [file Table1.DOCX]

Supplementary Material

The Changing Landscape of Neuroscience Research, 2006–2015: A Bibliometric Study

Andy Wai Kan Yeung^*^, Tazuko K. Goto, W. Keung Leung

*** Correspondence:** Andy Wai Kan Yeung: ndyeung@hku.hk

**Supplementary Table 1. List of core neuroscience journals from 2006–2015.**

| Journal title | 2006 | 2007 | 2008 | 2009 | 2010 | 2011 | 2012 | 2013 | 2014 | 2015 |
| --- | --- | --- | --- | --- | --- | --- | --- | --- | --- | --- |
| *Behavioural Brain Research* | • |  | • | • | • | • | • | • | • | • |
| *Brain Research* | • | • | • | • | • | • | • | • | • | • |
| *Cerebral Cortex* |  |  |  |  |  |  |  |  |  | • |
| *European Journal of Neuroscience* | • | • | • | • | • | • | • | • |  |  |
| *Experimental Brain Research* | • | • | • | • | • | • | • | • | • |  |
| *Frontiers in Behavioral Neuroscience* | NA |  |  |  |  |  |  |  | • | • |
| *Frontiers in Cellular Neuroscience* | NA |  |  |  |  |  |  |  | • | • |
| *Frontiers in Human Neuroscience* | NA | NA |  |  |  |  |  | • | • | • |
| *Frontiers in Neuroscience* | NA |  |  |  |  |  |  |  | • | • |
| *Human Brain Mapping* |  |  |  |  |  |  |  |  | • | • |
| *Journal of Alzheimer’s Disease* |  |  |  |  | • | • | • | • | • | • |
| *Journal of Clinical Neuroscience* |  |  |  | • | • | • | • | • | • | • |
| *Journal of Comparative Neurology* |  | • |  |  |  |  |  |  |  |  |
| *Journal of Neurochemistry* | • | • | • | • | • | • | • |  |  |  |
| *Journal of Neurophysiology* | • | • | • | • | • | • | • | • | • | • |
| *Journal of Neuroscience* | • | • | • | • | • | • | • | • | • | • |
| *Journal of Neuroscience Methods* |  |  | • | • | • | • |  |  |  |  |
| *Journal of Neuroscience Research* |  | • | • |  |  |  |  |  |  |  |
| *Journal of Physiology (London)* |  | • | • | • | • | • | • | • |  |  |
| *Journal of Stroke and Cerebrovascular Diseases* |  |  |  |  |  |  |  | • | • | • |
| *Journal of the Neurological Sciences* |  |  | • | • | • | • | • | • | • | • |
| *Neural Regeneration Research* |  |  |  |  |  | • | • | • |  |  |
| *Neurobiology of Aging* |  |  |  |  |  |  | • | • | • | • |
| *NeuroImage* | • | • | • | • | • | • | • | • | • | • |
| *Neuron* |  |  |  |  |  |  |  | • | • | • |
| *Neuropharmacology* |  |  |  |  |  |  | • | • | • | • |
| *Neuropsychologia* |  |  |  | • | • | • | • |  |  | • |
| *Neuroreport* |  | • |  |  |  |  |  |  |  |  |
| *Neuroscience* | • | • | • | • | • | • | • | • | • | • |
| *Neuroscience Letters* | • | • | • | • | • | • | • | • | • | • |
| *Pain* |  |  | • |  |  |  |  | • |  |  |
| *Psychopharmacology* |  |  |  | • |  | • | • | • | • | • |
| *Vision Research* | • |  |  |  |  |  |  |  |  |  |

A black dot denoted that journal belonged to the core journals (assigned to Zone 1 according to Bradford’s law) in that year. NA, non-applicable because that journal did not exist in that year. Core journals are the most productive journals; together they contributed to one-third of total count of articles and reviews in the census year (Table 4).
